# A comparison of dosimetric parameters in high-dose-rate intracavitary brachytherapy using tandem and ring applicators: is the combination of a rectal retractor and radio-opaque vaginal packing better than rectal retractor alone for rectum sparing in cervical cancer?

**DOI:** 10.3332/ecancer.2022.1439

**Published:** 2022-08-08

**Authors:** Siddharth Vats, Shabnum Thakur, Mukesh Sharma, Manish Gupta, Lalit Chandrakant, Muninder Negi, Swati Verma, Anup Negi, Vivek Kumar, Parul Sharma, Rattan Mahesh Negi

**Affiliations:** 1 Department of Radiotherapy & Oncology, IGMC Shimla, Himachal Pradesh, 171001, India; 2 Department of Radiotherapy & Oncology, Dr. YSP Govt. Medical College, Nahan, Himachal Pradesh, 173001, India; 3 Department of Radiotherapy & Oncology, RP Govt. Medical College, Tanda, Kangra, Himachal Pradesh, 176001, India; 4 Department of Radiotherapy & Oncology, AIIMS Rishikesh, Rishikesh, Uttarakhand, 249203, India

**Keywords:** intracavitary brachytherapy, high dose rate, cervical cancer, tandem and ring applicator, rectal sparing

## Abstract

**Purpose:**

To describe a novel packing technique of combining radio-opaque vaginal packing (VP) with rectal retractor (RR) blade to displace rectum and to compare the standard International Commission on Radiation Units & Measurements-38 (ICRU-38) rectal, bladder point doses and volume parameters with the conventional technique of using RR alone with tandem and ring (T&R) applicators in high-dose-rate (HDR) intracavitary brachytherapy (ICB) for cervical cancer.

**Materials and methods:**

A retrospective analysis of the treatment plans of a cohort of 24 consecutively treated patients with locally advanced cervical cancer was performed. All patients received external beam radiotherapy with a dose of 45–50.4 Gy, in fractions of 1.8–2 Gy, to the whole pelvis with concurrent weekly chemotherapy, followed by HDR-ICB. The treatment plans for each brachytherapy session using T&R applicators that these patients received were included for analysis. A total of 17 treatment plans in which the combination of RR and radio-opaque VP was used for rectum separation were included in the combination (RR+VP) group, and 21 treatment plans in which a RR alone was used were included in the conventional (RR) group.

**Results:**

The mean dose to the ICRU rectal point was 39.7% of the point A dose (range, 28.8%–50.4%; median 38.6%) in the RR+VP group and 52.7% (range, 46.9%–63.6%; median 51.7%) in the RR group. This 13% reduction in the mean dose to the ICRU rectal point was statistically significant (*p* = 0.001). The mean dose to the ICRU bladder point was 55.4% of the point A dose (range, 26.3%–73.6%; median 57.4%) in the RR+VP group and 45.3% (range, 23.7%–72.7%; median 46.9%) in the RR group. This 10.1% increase in the mean dose to the ICRU bladder point was also significant (*p* = 0.024). The other point dose and volume parameters were similar between groups.

**Conclusion:**

Compared to the conventional use of a RR blade alone for rectum separation, combining a RR blade with radio-opaque VP significantly reduced the ICRU rectal point dose.

## Introduction

Brachytherapy (BT) is an essential component of the definitive management of cervical cancer since it delivers high dose to the gross disease and allows a rapid dose fall off at the location of organs at risk (OARs). The optimal displacement of OARs from the applicators decreases the risk of toxicities. The International Commission on Radiation Units & Measurements (ICRU) Report 38 has precisely codified the determination of absorbed doses at ICRU rectal, bladder points and reference volumes to standardise the reporting of intracavitary brachytherapy (ICB) [1, 2].

Our institutional experience was only with tandem and ovoid (T&O) applicators, and we were using radio-opaque vaginal packing (VP) in all BT sessions for rectum and bladder displacement. With the introduction of high-dose-rate (HDR) BT, we started using tandem and ring (T&R) applicators with rectal retractor (RR) blades. Due to concerns about long-term rectal toxicity, we tried to ensure that the rectal dose comprised a small fraction of the treatment dose [3–5]. Many studies have established a clear correlation between the ICRU rectal point doses D_ICRU_, D_2cc_, D_1cc_ and D_0.1cc_ and late rectal toxicity [6–8]. During the transition from low-dose-rate to HDR, it was desirable to further reduce the percentage of the prescribed dose that reached OARs by increasing the distance between the OARs and applicators [9]. Various studies have shown that the use of RR leads to significantly lower rectal dose parameters than the use of VP or a tandem Foley balloon (FB) in HDR ICB with T&R applicators [10, 11]. In the case of VP, the amount of radio-opaque VP required to adequately fill the space remaining in the vaginal cavity after applicator placement to achieve optimal displacement of the OARs varies from one patient to another. However, with vendor-supplied RR blades, a fixed set is used in all patients. Nonetheless, ICRU 89 report also mentions that RR blades can be used in addition to VP [1]. We are not aware of any study that combines an RR blade with radio-opaque VP to displace the rectum away from radiation sources and compares the rectal doses between one or both standard methods of rectal retraction, the RR blade or VP, in HDR ICB using T&R applicators. We logically modified the conventional BT application procedure for using T&R applicators by deliberately placing radio-opaque VP, posterior and cranial to the RR blade, to further retract the rectum away from radiation sources. VP is achieved with a thin ribbon, and the extent and amount of packing can be customised for a range of spaces and to different patients. Gradually, in all BT procedures, in which the patient’s anatomy allowed for the placement of T&R applicators with RR blades, we started combining radio-opaque VP with an RR blade. We accumulated our institutional experience of combining an RR blade with radio-opaque VP in HDR-ICB using T&R applicators and retrospectively analysed the data in this observational study. The primary objective was to compare the standard ICRU-38 rectal point doses and other dose-volume parameters between patients consecutively treated using a combination of a rectal retractor blade and radio-opaque vaginal packing (RR+VP) and those treated with a RR blade alone.

## Material and methods

### Patients

The institutional records of locally advanced cervical cancer patients registered in our department between November 2017 and August 2018, who completed external beam radiotherapy (EBRT) and had an adequate tumour response to receive BT were reviewed. This yielded a cohort of 24 patients who received at least one fraction via T&R applicators. The treatment plans of these patients with standard loading patterns and reference points prescribed as per the ICRU-38 report were considered eligible for analysis. Patients treated with plans that deviated from the ICRU-38 guidelines, with suboptimal placement of the applicators due to a learning curve or with suboptimal dose prescriptions were not eligible for analysis.

### Brachytherapy procedure and planning

The BT procedure was carried out under intravenous sedation and analgesia in all patients as per the institutional protocol. Both T&O and T&R applicators were used according to physician preference and logistic accessibility to the applicator sets. After inserting the central tandem, the largest diameter ring that the upper vagina could accommodate was chosen and threaded over the tandem. The T&R applicator was positioned to encircle the cervix and was pushed against the cervix. While the ring was kept in position, the available space posterior and superior to the ring in the upper vagina was visualised to assess its capacity for accommodating a RR and additional VP. The RR was placed and clamped with an interlock system. Standard gauze packing soaked in normal saline was used to stabilise the applicator and to push the bladder anteriorly in all sessions. The above procedure in which an RR alone was used for rectum separation is referred to as the conventional procedure in this paper. We developed a combination/modified insertion procedure in which all the steps described above for the conventional procedure remained the same, but in addition to the above steps, we deliberately placed radio-opaque VP in addition to the RR blade in the vaginal fornices and along the upper part of the posterior vaginal wall, as per the available capacity, without overstretching the mucosa to avoid mucosal tears. After the procedure, orthogonal radiographs were taken by a conventional simulator (Varian, Acuity, USA), and the images were transferred to the treatment planning system via a digital imaging and communication in medicine connection.

ICB treatment planning was performed on the treatment planning system Oncentra Brachy v4.6 and was delivered by Nucletron, MicroSelectron HDR V3 (18-channel) remote after-loading machine using Ir-192. The orthogonal images taken with the applicators in place were reconstructed, and source positions were loaded according to standard loading pattern following the Manchester system. Point A was taken as 2 cm superior to the lower most intrauterine source along the axis of the intrauterine tandem and 2 cm lateral in the plane of the uterus. A fixed step size of 2.5 mm was used for all applications. The dwell times were equal for all dwell positions. Only lateral dwell positions in the ring were activated. The doses to the ICRU rectal point, bladder point and point B were taken according to the recommendations of the ICRU-38 and reported per insertion in all cases.

Even with the advent of 3-D image-based BT, these points are still relevant, as many centres in low- and middle-income countries are still prescribing doses to point A, and studies comparing absorbed doses at ICRU reference points with CT-based treatment planning show that the ICRU rectal point dose correlates well with D2cc [12–14].

### Dosimetric calculations

The treatment plans of all patients were reviewed retrospectively. The following parameters were studied for all plans: tandem length, tandem angle and ring size of the applicator used during a particular session of ICB. The dwell positions along the central tandem and in the ring were noted for each individual fraction. The accuracy of the prescribed point A, point B, rectal point and bladder points was verified. As the absolute dose prescribed to point A is different in the three regimens, it is normalised to 100% to simplify the data analysis. For the same reason, the absolute doses to point B and the ICRU rectal and bladder points were also expressed as a percentage of the prescription dose. We measured a pear-shaped 100% isodose reference volume (Vol_ref_) for each ICB session and described the volume in terms of its three maximal dimensions: height (d_h_) measured in an oblique frontal plane along the intrauterine sources, width (d_w_) measured in the same plane perpendicular to the intrauterine sources and thickness (d_t_) measured in the oblique sagittal plane perpendicular to the intrauterine sources. Vol_ref_ was calculated as the product of d_h_ × d_w_ × d_t_ [2]. Similarly, H, W and T were applied to the same parameters of a 60 Gy reference volume (Vol_60Gy_), which was defined as the 60 Gy isodose surface resulting from the additional dose contribution from whole pelvic irradiation and all intracavitary insertions [2]. Applying the linear-quadratic model and an alpha/beta ratio of 10 Gy for the tumour effect, the isodose curve, which encloses the 60 Gy volume for patients who received 50 Gy EBRT followed by two sessions of 9 Gy each, was represented by 3.15 Gy. Similarly, the 60 Gy isodose volume was enclosed by 2.35 Gy in patients who received 50 Gy EBRT followed by three sessions of 7 Gy each. The total reference air kerma (TRAK) values for each fraction were also recorded.

### Statistical analysis

Baseline characteristics, stage and applicator parameters were compared with X^2^ tests. Mean values and standard deviations were calculated for 21 applications in the conventional (RR) group and 17 in the combination (RR+VP) group. These mean values were then compared between the two groups with independent sample *t* tests. Paired sample *t* tests were used for subset analysis. All statistical analyses were performed using IBM Corp. Released 2019. IBM SPSS Statistics for Windows, Version 26.0. Armonk, NY: IBM Corp. A *p* value < 0.05 was considered significant.

## Results

A total of 17 women received two fractions of 9 Gy each, four women received three fractions of 7 Gy each, one woman received four fractions of 7 Gy each and two received two fractions of 9.5 Gy each. This amounted to 54 HDR ICB sessions in this cohort of 24 patients. Nine ICB sessions delivered using T&O applicators were excluded. A total of 45 ICB sessions were delivered using T&R applicators. Seven fractions out of 45 were not eligible for analysis because of the following reasons: the prescribed dose was too low in two treatment plans; suboptimal applicator placement in two sessions and the standard loading patterns and ICRU-38 recommendations were not followed in three sessions. This left 38 ICB sessions for analysis. Out of these 38 sessions, 21 ICB sessions performed using an RR alone constituted the RR group, and 17 ICB sessions, in which an RR blade was combined with radio-opaque VP, constituted the RR+VP group.

### Patient characteristics

Both groups were statistically balanced with regard to the distribution of variables, such as stage and applicator characteristics, which can influence dose-volume parameters (Table 1).

### Dosimetric parameters

The comparison between dose and volume parameters is shown in Table 2. For the same dose prescribed to point A in both groups, the mean dose to the ICRU rectal point was 39.7% of the prescription dose (range, 28.8%–50.3%; median 38.6%) in the RR+VP group and 52.7% (range, 46.9%–63.6%; median 51.6%) in the RR group. There was a 13% reduction in the mean dose to the ICRU rectal point (*p* = 0.001). The mean dose to the ICRU bladder point was 55.4% of the prescription dose (range, 26.3%–73.6%; median 57.4%) in the RR+VP group and 45.3% (range, 23.6%–72.7%; median 46.9%) in the RR group. There was a 10.1% increase in the mean dose to the ICRU bladder point (*p* = 0.024). The mean dose to point B was similar at 22.7% of the prescription dose (range, 20.4%–24.1%; median 22.8%) in the RR+VP group and 22.8% (range, 20.1%–25.2%; median 23.0%) in the RR group. The mean reference volume (Vol_ref_) for the BT part of treatment expressed as d_h_ × d_w_ ×d_t_ was similar at 142.2 cc (range, 107.6–195.7 cc; median 146.0 cc) in the RR+VP group and 143.7 cc (range, 103.4–197.8 cc; median 149.9 cc) in the RR group. The mean 60 Gy reference volume (Vol_60Gy_) expressed as H×W×T was similar at 557.2 cc (range, 326.3–703.0 cc; median 582.3 cc) in RR+VP group and 582.99 cc (range, 327.6 cc–721.0 cc; median 616.3 cc) in the RR group. The mean TRAK value (Gy/hour at 1 m) per application was also similar at 0.00446 (range, 0.00339–0.00566; median 0.00455) in the RR+VP group and 0.00476 (range, 0.00349–0.00561; median 0.00498) in the RR group.

### Subset analysis

**Figure 1.**
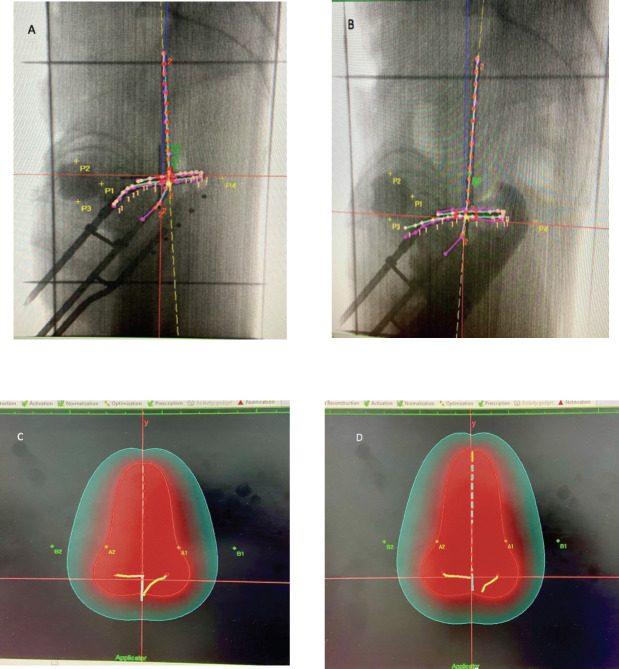
Planning images of one patient who completed 50 Gy EBRT, followed by three fractions of 7 Gy HDR BT using the same tandem ring applicator with a tandem length of 6 cm, angle of 45^°^ and a ring size of 3 cm during two consecutive fractions. (A) Applicator placement during the first fraction performed using a RR alone for rectal separation. P1 = ICRU bladder point, P4 = ICRU rectal point. (B) The same applicator placement during the second fraction using a combination of rectal retractor and radio-opaque vaginal packing (RR + VP). P1 = ICRU bladder point, P4 = ICRU rectal point. (C) During the first fraction delivered with RR alone, a pear-shaped reference volume (Vref) enclosed within a 7 Gy (100%) red isodose curve was 149.10 cc (d_h_ × d_w_ × d_t_) and that a 60 Gy reference volume (V60Gy) represented by a 2.4 Gy (33.6% of 7 Gy) green isodose curve in this case was 643.1 cc (H × W × T) (see text details on measuring the dimensions and calculating the 60 Gy volume). The point B dose was 1.6 Gy (22.7% of point A dose), ICRU rectal point dose was 4.17 Gy (59.6% of point A), ICRU bladder point dose was 2.5 Gy (35.3% of point A) and TRAK value was 0.00392 Gy. (D) During the second fraction delivered using the same applicator parameters but a combination of RR + VP for rectal separation, the reference volume (Vref) enclosed within a 7 Gy (100%) red isodose curve was 156.6 cc (d_h_ × d_w_ × d_t_), and the 60 Gy reference volume (V60Gy) enclosed by a 2.4 Gy green isodose curve was 682.4 cc (H × W × T). The point B dose was 1.6 Gy (23.8% of point A dose), the ICRU rectal point dose was 2.7 Gy (38.2% of point A), the ICRU bladder point dose was 3.7 Gy (52.9% of point A) and the TRAK value was 0.00406 Gy. In this patient, the BT fraction delivering the same prescribed dose to point A and treating equal 60 Gy isodose volumes, using a rectal retractor in combination with radio-opaque vaginal packing (RR+VP) reduced the dose to the ICRU rectal point by 1.5 Gy (21.4%) compared to the fraction delivered using a RR alone.

Patients differed between the two comparison groups. The individual anatomical variations among patients may be a possible confounding factor. However, when two methods, i.e., the combination of RR+VP and RR alone, were applied to the same patient during different insertions, patients could act as their own internal control, thus eliminating this possible confounding effect. In an attempt to account for this, subset analysis of ICB fractions was performed on five such women who received at least one fraction with each method. In these women, the ICB sessions that were done using the same tandem length, angle and ring size during both the combination procedure (RR+VP) and the conventional procedure (RR alone) were compared. This subset analysis was performed as an intrapatient comparison, and the results are presented in Table 3. For the same dose prescribed to point A in both groups, the mean dose to the ICRU rectal point was 40.5% of the prescription dose in the RR+VP group and 55.5% in the RR group. There was a 15.0% reduction in the mean dose to the ICRU rectal point (*p* = 0.013). The mean dose to the ICRU bladder point was 59.9% of the prescription dose in the RR+VP group and 46.5% in the RR group. There was a 13.4% increase in the mean dose to the ICRU bladder point (*p* = 0.019). The mean point B doses, mean reference volumes (V_ref_) for the BT part of treatment, mean 60 Gy reference volumes (V_60Gy_) and mean total TRAK values were similar in both groups. Figure 1 shows simulation and planning images of the fractions received by the same patient with the two methods of rectum separation.

## Discussion

T&O applicators with VP and T&R applicators with RR are the two most commonly used applicators for ICB in cervical cancer [15, 16]. T&R applicators have recently become popular in image-guided BT (IGBT) for cervical cancer due to their reproducible geometry and ease of application compared to T&O applicators [17, 18]. Studies have shown that in dosimetric comparisons between the two applicators, T&O applicators treat larger volumes than T&R applicators [19, 20]. In terms of rectal dose, T&R applicators with an RR achieve significantly better rectum sparing than T&O applicators with VP when the reference doses were prescribed as per the Groupe Européen de Curiethérapie-European Society for Radiotherapy & Oncology (GEC-ESTRO) guidelines [17]. In the EMBRACE (European study on MRI-guided brachytherapy in locally advanced cervical cancer) 1 study, patients treated with T&R applicators showed a more favourable therapeutic ratio in the evaluation of target, bladder and rectum doses and V_85Gy_ [21]. Kong *et al* [11] compared three rectal retractor methods in terms of doses to OARs and concluded that compared with a tandem FB and VP, T&R applicators with an RR significantly reduced the dose to the rectum and sigmoid. Gaudet *et al* [10] also concluded that the use of RR blades resulted in a significant reduction in rectal dose compared to the use of VP in BT sessions performed using T&R applicators.

In the abovementioned studies, T&R applicators with an RR were used in all patients who were eligible for ICB and had suitable anatomy, with the parametrium regressed to one-third or less after the completion of EBRT. There is a subgroup of patients within this larger patient population who are suitable for ICB but have asymmetric, superficial or obliterated vaginal fornices and a narrow vaginal cavity. These patients are considered to be ideal patients for T&R applicators [18, 22]. However, the use of T&R applicators is not restricted to this subgroup of patients in most centres. The studies discussed above affirm that the conventional ICB approach of using T&R applicators, in which a fixed RR is used for rectum separation and saline-soaked VP is placed in the centre for stabilisation and anteriorly for bladder separation, is the best method for rectum sparing. However, in this study, when we used T&R applicators with a modified approach of combining radio-opaque VP with a fixed RR blade, the average reduction in the mean dose to the ICRU rectal point (expressed as % of point A dose) was 13.03% per fraction as compared to the conventional approach of using RR alone (*p* = 0.001). In the subset analysis, this average reduction in rectal dose per fraction was 15% (*p* = 0.013).

It appears that the vendor-supplied RR blade is of a standard size for all patients, whereas the capacity of vaginal fornices varies from patient to patient. At one end, there are patients with roomy vagina/fornices, and at the other end, there are patients with obliterated/absent vaginal fornices or a narrow vagina. The remaining space in the fornices, after placement of the RR blade, especially in the case of roomy vagina, becomes inaccessible for packing after the RR blade is assembled. For this reason, in conventional procedures performed using T&R applicators with RR blades, VP is possible only in the anterior and centre, but not posteriorly, as the posterior space is occupied by the RR blade. We could deliberately place radio-opaque VP based on the available space in the fornices, approaching from the posterior aspect of the RR blade without overstretching to avoid mucosal tears. The goal was to increase rectum separation and hence, a greater reduction in rectal dose. The same can be achieved by using traditional VP instead of a RR blade since there will be no obstruction due to the presence of the RR blade, and the amount of packing can be customised as per the patient’s anatomy. This could be a possible reason for the rectal doses being significantly lower with gauze packing than with RR blades in the study by Patil *et al* [23], which differed from the studies by Kong *et al* [11] and Gaudet *et al* [10] that were in favour of the RR blade.

It is pertinent to discuss similar packing methods used in place of conventional VP to displace OARs with T&O applicators [24–28]. Rockey *et al* [24] showed that the commercially available vaginal balloon packing system provides statistically equivalent OAR protection compared to traditional VP in HDR-BT performed using T&O applicators. Due to the major benefit of reproducibility among fractions and minimum reliance on technique, balloon packing was suggested for low-volume centres with little experience in gauze packing [24]. Rai *et al* compared bladder and rectum doses using a standard size, bladder-rectum spacer balloon (BRSB) with standard gauze packing [25, 26]. The BRSB resulted in significant reductions in both the dose and interfraction dose variation for D_2cc_ of the rectum, but no significant reduction in dose variation to other sub-volumes of the bladder and rectum was seen. The authors recommended the use of a BRSB when there is limited physician expertise or if general anaesthesia is not routinely used. In patients with a narrow vagina, a non-transparent balloon obscured the visualisation of the external os and crowded inside the vagina due to oversizing, which made the applications difficult and time consuming. To use a BRSB in patients with a narrow vagina, the authors suggested transparent balloons of variable sizes [25, 26]. Sud *et al* [27] compared speculum-based VP (SBVP) with conventional gauze packing during tandem and ovoid HDR-ICB. In patients with appropriate anatomy, improved bladder sparing and comparable rectum sparing, relative to that achieved with gauze packing, could be achieved. However, in patients requiring dose optimisation, SBVP did not differ from conventional gauze for bladder sparing and was associated with significant increases in rectal dose volume parameters. The authors found that the insertion of the second ovoid through the speculum to be challenging in the narrow vaginal canals [27]. However, when Eng *et al* [28] used intravaginal FBs in addition to conventional packing, a significant dose reduction to the bladder and rectum could be achieved due to further displacement of these structures. They insisted that proper VP is crucial and that the addition of FBs to properly placed conventional packing helped to reduce the doses to the rectum and bladder. One challenge is that vaginal balloons are tedious to craft in-house, thus increasing the time costs for medical professionals, and the customised packing of this method imparts a degree of operator dependence [28].

The combination procedure (RR+VP) is less time-consuming, although it requires skill and acumen from the physician. This approach provides a new option in the debate of the best method for rectum sparing by further increasing the retraction achieved with the RR blade, challenging the preference of VP over an RR blade as the standard method for rectal retraction with T&R applicators. No comparison of the combination procedure (RR+VP) with either of the two standard methods of rectal retraction, i.e., RR alone or VP alone, for rectum separation has been reported in the literature. Another finding in our study is that the decrease in rectal dose is associated with an increase in bladder dose. Earlier in the literature, it was suggested that the insertion of an RR may actually displace the applicators towards the bladder, making anterior VP even more important [15]. Our study reiterates this point, but a prospective study with a larger sample size would answer whether this simultaneous increase in bladder dose can be avoided with careful anterior packing. Although our study results were obtained based on the standard ICRU-38 rectum and bladder points, the findings have relevance for 3-D image-based BT, as other studies comparing absorbed doses at ICRU reference points with CT-based treatment planning show that the ICRU rectal point dose correlates well with D2cc, while the ICRU bladder point underestimates D2cc [14]. This is also supported by the fact that the contemporary dose limit to the rectum (D_2cc_) does not differ from the standard ICRU rectal point dose and is <75 Gy, whereas the bladder (D_2cc_) dose limit is high and in the range of 90 Gy, compared to the ICRU bladder point dose of <80 Gy [29]. Moreover, bladder complications remain a minor problem in HDR cervical cancer treatment compared to the rectal complications [15]. Cervical carcinoma has a high prevalence in regions with fewer resources, which are already at a disadvantage due to a lack of preventive screening. The availability of 3-D image-based BT techniques in many regions remains limited due to the high costs, need for sophisticated planning software and lack of trained personnel. In a survey from India, magnetic resonance imaging-based IGBT was performed by only 9% of the responding institutes in 2019 [12]. Similarly, in another survey, it was seen that although the delineation of OARs based on computed tomography was done routinely, target volume delineation and dose prescription/optimisation for the same were performed only in a handful of centres (5 out of 57), as the majority prescribe a dose to point A [13]. Vast experience has accumulated in the use of 2-D techniques in the previous decades, and the rational implementation of such techniques has been recently suggested to optimise the utilisation of available resources for achieving the best possible clinical outcome [30].

This study was retrospective, and the allocation of patients and applicators chosen during the BT sessions in the two groups was beyond the control of the authors. Patients in both groups had also received one or two BT sessions using T&O applicators; therefore, it was not possible to evaluate differences in survival and side effects. We are planning a prospective study with a large number of patients and 3-D treatment planning to compare the combination of RR+VP with standard methods of rectal retraction, i.e., RR blade alone and VP alone, in the same patient for different insertions. This study will also address the potential risk for increases in the incidence of discomfort and mucosal tears due to possible overstretching of the vaginal mucosa.

## Conclusion

The combination of a RR blade and radio-opaque VP leads to a significant reduction in dose to ICRU rectal point compared to the use of a RR blade alone in HDR ICB for cervical cancer performed using T&R applicators.

## Conflicts of interest

The authors report no conflicts of interest.

## Funding

No funding was received for this study.

## Figures and Tables

**Table 1.** Distribution of stage- and applicator-related characteristics in the two groups.

Characteristic	Number	*p* value (x^2^)
Patients RR group RR+VP group	16 13	
FIGO stage Stage I RR RR+VP Stage II RR RR+VP Stage III RR RR+VP Stage 4 RR RR+VP	1 2 11 5 4 5 0 1	0.331
Insertion plan RR RR+VP	21 17	
Tandem length-6 cm RR RR+VP Tandem length-4 cm RR RR+VP Tandem length-2 cm RR RR+VP	14 8 5 8 2 1	0.332
Tandem angle-30^°^ RR RR+VP Tandem angle-45^°^ RR RR+VP	9 10 10 7	0.691
Ring size -30 mm RR RR+VP **-**26 mm RR RR+VP	15 12 6 5	0.955

RR, Rectal retractor group; RR+VP, Rectal retractor along with radio-opaque vaginal packing group

**Table 2.** Comparison of dose and volume parameters between the two groups.

Physical parameter	*N* (fractions)	Mean	SD	*p* value^a^
Point B dose (% of prescription) RR RR+VP	21 17	22.8 22.7	1.4 1.1	0.795
ICRU rectal point dose (% of prescription) RR RR+VP	21 17	52.7 39.7	4.9 8.1	***0.001***
ICRU bladder point dose (% of prescription) RR RR+VP	21 17	45.3 55.4	12.6 13.8	***0.024***
ICRU reference volume (V_ref_) (d_h_ × d_w_ × d_t_) in cc RR RR+VP	21 17	143.8 142.2	23.9 23.3	0.844
ICRU 60 Gy volume (V_60Gy_) (H × W × T) in cc RR RR+VP	21 17	582.9 557.2	103.6 131.8	0.504
TRAK value RR RR+VP	21 17	0.00476 0.00446	0.00063 0.00065	0.844

RR, Rectal retractor group; RR+VP, Rectal retractor along with radio-opaque vaginal packing group; ICRU, International Commission on Radiation Units & Measurements; CI, Confidence interval; SD, Standard deviation

a Statistically significant *p* values are shown in bold italic print

**Table 3.** Comparison of rectal retractor alone (RR) versus the combination of rectal retractor with vaginal packing (RR+VP) in a subset of patients who received HDR fractions with both methods of rectum separation for different insertions.

Parameters		Patient 1	Patient 2	Patient 3	Patient 4	Patient 5	Mean	Standard Deviation	*p* value^a^
**Stage**		III-B	II-B	II-B	I-B	II-B	NA	NA	NA
**Fraction included**	RR RR+VP	2_nd_ 1_st_	2_nd_ 3_rd_	3rd 2_nd_	1_st_ 2_nd_	3_rd_ 2_nd_	NA NA	NA NA	NA NA
**Tandem length**	RR RR+VP	4 4	6 6	4 4	6 6	6 6	NA NA	NA NA	NA NA
**Tandem angle**	RR RR+VP	45^°^ 45^°^	45^°^ 45^°^	30^°^ 30^°^	45^°^ 45^°^	30^°^ 30^°^	NA NA	NA NA	NA NA
**Ring size (cm)**	RR RR+VP	2.6 2.6	3 3	2.6 2.6	3 3	3 3	NA NA	NA NA	NA NA
**Point A dose** ** (%)**	RR RR+VP	100 100	100 100	100 100	100 100	100 100	100 100	NA NA	NA NA
**Point B dose** **(% of prescription)**	RR RR+VP	20.1 22	22.7 23.8	21.4 21.4	23 23.5	24.5 23.7	22.3 22.9	1.7 1.1	0.307
**ICRU rectal point dose** **(% of prescription)**	RR RR+VP	63.5 49.5	59.6 38.2	52.8 50.3	54.9 32.6	46.9 31.8	55.5 40.5	6.4 8.9	***0.013***
**ICRU bladder point dose(% of prescription)**	RR RR+VP	57.9 67.9	35.3 52.8	47.1 57.4	28.3 53.2	63.6 68.1	46.5 59.9	14.8 7.6	***0.019***
**V_ref_ (cc)**	RR RR+VP	107.1 122.9	149.9 156.7	119.7 121.7	152.9 156.7	164.0 152.6	138.7 142.1	24.2 18.1	0.487
**V_60Gy_ (cc)**	RR RR+VP	446.9 519.3	643.1 682.4	327.7 332.5	615.1 645.3	721.3 677.9	550.83 571.46	159.9 149.0	0.346
**TRAK value**	RR RR+VP	0.00416 0.00357	0.00342 0.00406	0.00349 0.00352	0.00503 0.00524	0.00427 0.00407	0.00412 0.00405	0.00075 0.00073	0.745

RR, Rectal retractor group; RR+VP, Rectal retractor along with radio-opaque vaginal packing group; ICRU, International Commission on Radiation Units & Measurements; NA, Not applicable

a Statistically significant *p* values are shown in bold italic print; *p* value of 0.05 is significant

## References

[1] (2013). Brachytherapy techniques and systems. J ICRU.

[2] International Commission on Radiation Units and Measurements (ICRU) (1985). ICRU Report 38, Dose and Volume Specification for Reporting Intracavitary Brachytherapy in Gynecology.

[3] Halperin EC, Wazer DE, Perez CA (2019). Principles and Practice of Radiation Oncology.

[4] Brenner DJ, Huang Y, Hall EJ (1991). Fractionated high dose-rate versus low dose-rate regimens for intracavitary brachytherapy of the cervix: equivalent regimens for combined brachytherapy and external irradiation. Int J Radiat Oncol Biol Phys.

[5] Brenner DJ, Hall EJ (1991). Fractionated high dose-rate versus low dose-rate brachytherapy of the cervix. I. General cosiderations based on radiobiology. Br J Radiol.

[6] Georg P, Kirisits C, Goldner G (2009). Correlation of dose-volume parameters, endoscopic and clinical rectal side effects in cervix cancer patients treated with definitive radiotherapy including MRI-based brachytherapy. Radiother Oncol.

[7] Koom WS, Sohn DK, Kim JY (2007). Computed tomography-based high-dose-rate intracavitary brachytherapy for uterine cervical cancer: preliminary demonstration of correlation between dose-volume parameters and rectal mucosal changes observed by flexible sigmoidoscopy. Int J Radiat Oncol Biol Phys.

[8] Mazeron R, Fokdal LU, Kirchheiner K (2016). Dose-volume effect relationships for late rectal morbidity in patients treated with chemoradiation and MRI-guided adaptive brachytherapy for locally advanced cervical cancer: results from the prospective multicenter EMBRACE study. Radiother Oncol.

[9] Orton CG, Somnay A (1991). Comparison of high and low dose rate remote afterloading for cervix cancer and the importance of fractionation. Int J Radiat Oncol Biol Phy.

[10] Gaudet M, Lim P, Yuen C (2014). Comparative analysis of rectal dose parameters in image-guided high-dose-rate brachytherapy for cervical cancer with and without a rectal retractor. Brachytherapy.

[11] Kong I, Vorunganti S, Patel M (2016). Prospective comparison of rectal dose reduction during intracavitary brachytherapy for cervical cancer using three rectal retraction techniques. Brachytherapy.

[12] Chatterjee A, Grover S, Gurram L (2019). Patterns of cervical cancer brachytherapy in India: results of an online survey supported by the Indian Brachytherapy Society. J Contemp Brachytherapy.

[13] Bandyopadhyay A, Basu P, Roy K (2018). Treatment of locally advanced carcinoma cervix with special emphasis on brachytherapy: a practice pattern survey among young radiation oncologist of India. South Asian J Cancer.

[14] Jamema SV, Saju S, Mahantshetty U (2008). Dosimetric evaluation of rectum and bladder using image-based CT planning and orthogonal radiographs with ICRU 38 recommendations in intracavitary brachytherapy. J Med Phys.

[15] Nag S, Erickson B, Thomadsen B (2000). The American Brachytherapy Society recommendations for high-dose-rate brachytherapy for carcinoma of the cervix. Int J Radiat Oncol Biol Phys.

[16] Gursel SB, Serarslan A, Meydan AD (2020). A comparison of tandem ring and tandem ovoid treatment as a curative brachytherapy component for cervical cancer. J Contemp Brachytherapy.

[17] Biltekin F, Gultekin M, Yilmaz MT (2010). Dosimetric comparison of two different applicators and rectal retraction methods used in inverse optimization-based intracavitary brachytherapy for cervical cancer. J Contemp Brachytherapy.

[18] Viswanathan AN, Beriwal S, De Los Santos JF (2012). American Brachytherapy Society consensus guidelines for locally advanced carcinoma of the cervix. Part II: high-dose-rate brachytherapy. Brachytherapy.

[19] Ma JK, Mourad WF, Allbright R (2015). Short-term clinical outcome and dosimetric comparison of tandem and ring versus tandem and ovoids intracavitary applicators. J Contemp Brachytherapy.

[20] Levin D, Menhel J, Rabin T (2008). Dosimetric comparison of tandem and Ovoids vs. tandem and ring for intracavitary gynecologic applications. Med Dosim.

[21] Serban M, Kirisits C, Leeuw A (2020). Ring versus ovoids and intracavitary versus intracavitary-interstitial applicators in cervical cancer brachytherapy: results from the EMBRACE I Study. Int J Radiat Oncol Biol Phys.

[22] Biltekin F (2019). Physical and dosimetric properties of the applicators used in cervix cancer brachytherapy: ICRU 89 recommendations. Turk J Oncol.

[23] Patil NG, Murthy V, Gupta T (2006). HDR brachytherapy in carcinoma cervix: rectal retractor or gauze packing?. Brachytherapy.

[24] Rockey WM, Bhatia SK, Jacobson GM (2013). The dosimetric impact of vaginal balloon-packing on intracavitary high-dose-rate brachytherapy for gynaecological cancer. J Contemp Brachyther.

[25] Rai B, Patel FD, Chakraborty S (2013). Bladder-rectum spacer balloon in high-dose-rate brachytherapy in cervix carcinoma. Int J Radiation Oncol Biol Phys.

[26] Rai B, Patel FD, Chakraborty S (2015). Bladder-rectum spacer balloon versus vaginal gauze packing in high dose rate brachytherapy in cervical cancer: a randomized study (part 2). Clin Oncol.

[27] Sud S, Roth T, Jones E (2018). Clinical analysis of speculum-based vaginal packing for high-dose-rate intracavitary tandem and ovoid brachytherapy in cervical cancer. J Contemp Brachyther.

[28] Eng TY, Patel AJ, Ha CS (2016). Rectal and bladder dose reduction with the addition of intravaginal balloons to vaginal packing in intracavitary brachytherapy for cervical cancer. Brachytherapy.

[29] Haie-Meder C, Dumas I, Paumier A (2008). Implementation of GEC-ESTRO recommendations on 3-D based image brachytherapy. Cancer Radiother.

[30] Gupta A, Dey T, Rai B (2021). Point-based brachytherapy in cervical cancer with limited residual disease: a low- and middle-income country experience in the era of magnetic resonance-guided adaptive brachytherapy. JCO Glob Oncol.

